# Qualitative and quantitative analysis of chemical constituents in goupi plaster prepared by various extraction methods using UPLC-Q-Exactive-MS and UPLC-MS/MS

**DOI:** 10.1016/j.heliyon.2024.e31365

**Published:** 2024-05-18

**Authors:** Tong Guan, Rong Wang, Jiajing Wang, Qingqing Zhang, Ziheng Liu, Zhixin Yang, Feng Guan, Weinan Li, Yanhong Wang

**Affiliations:** a School of Pharmacy, Heilongjiang University of Chinese Medicine, Harbin, China; b The Second Affiliated Hospital of Heilongjiang University of Traditional Chinese Medicine, Harbin, China

**Keywords:** Goupi plaster, Extraction method, Qualitative, Quantitative

## Abstract

Goupi plaster, a representative preparation of black plaster, has demonstrated promising effects in treating knee osteoarthritis. However, high temperature used in traditional frying extraction may cause decomposition of its effective components, thus limiting the efficacy. This study aimed to explore the scientific nature of the traditional preparation technology of Goupi plaster, and to compare the effects of different extraction methods on the types of chemical components and the content of index components. The UPLC-Q-Exactive-MS and UPLC-MS/MS technologies which have high efficiency, sensitivity and accuracy, were used to qualitatively and quantitatively analyze the chemical components of Goupi plaster under different preparation processes. The results show that the extraction solvent approach is different from the traditional frying extraction method, and has a positive effect. However, the mechanism of action of Goupi plaster is complex and its pharmacological effects are diverse. Future studies should explore whether it necessary to change the frying extraction method. This experiment provides a theoretical basis that will guide further scientific discussion and research into the frying extraction of Goupi plaster.

## Highlights

• The effect of different extraction methods on chemical constituents of Goupi plaster were explored.
• Three index components of Goupi plaster were determined by cleavage pathway.
• It is verified that the preparation process of Goupi plaster was related to the chemical composition.

## 1 Introduction

Plaster is one of the five traditional dosage forms of traditional Chinese medicine. Black plaster refers to a plaster made from medicinal materials, vegetable oil and red lead, which is spread on a mounting material and applied to the skin for external use. It has the advantages of good operability and safety profile [1]. Goupi plaster is one of the main preparations of the traditional Chinese medicine classic formula black plaster, which has been continuously improved and applied since 1963 in Chinese pharmacopoeia. It is mainly composed of 29 kinds of Chinese medicinal materials, including *Radix Aconiti*, *Radix Aconiti Kusnezoffii*, *Notopterygii Rhizoma*, etc. Goupi plaster can improve and treat knee osteoarthritis, frozen shoulder, and other diseases. Currently, China has developed polices to improve the generation transmission and development of traditional Chinese medicine. As an important traditional Chinese medicine preparation, the application of Goupi plaster is often questioned because of its traditional techniques such as explosives and oil refining. The chemical composition of medicinal materials is complex, and high-temperature frying may cause damage to heat-sensitive components and insufficient extraction. Moreover, there is no record of quality control standards in the 2020 version of the “Chinese Pharmacopoeia”. The current quality control only requires that it undergoes softening point check and weight difference check. These challenges have significantly limited the future development of Goupi plaster. Therefore, comparing different extraction methods and screening the effective chemical components of Goupi plaster using modern extraction technology is an effective approach for verifying the scientificity of the traditional frying process [[2], [3], [4], [5], [6], [7], [8]].

In this study, we aimed to analyze the chemical components of Goupi plaster extracted by different solvents and determine the content of some components. We employed UPLC-Q-Exactive-MS technology to qualitatively analyze the chemical components in Goupi plaster, and the UPLC-MS/MS technology to quantitatively analyze three representative components in Goupi plaster. Our research results indicate that the change of extraction solvent is different from that of traditional frying extraction method, and this effect is positive. This study provides theoretical basis to guide future research and development of Goupi plaster.

## 2 Materials and methods

### 2.1 Chemicals and herbal materials

The standard compounds of sinomenine, cinnamaldehyde, and osthole were purchased from China Food and Drug Testing Institute (Beijing, China). Soybean oil was obtained from Lu Hua Group (Shandong, China), while distilled water was purchased from Watsons Food & Beverage Company (Guangzhou, China). The LC-MS grade methanol and LC-MS grade acetonitrile were both purchased from Merck (Merck, USA). The LC-MS formic acid was purchased from West Asia Reagent (Chengdu, China), while petroleum ether was purchased from Fuyu Fine Chemical Industry Group (Tianjin, China).

The Chinese medicinal herbs that make up the Goupi plaster, include *Radix Aconiti*, *Radix Aconiti Kusnezoffii*, *Notopterygium incisum*, *Angelicae Pubescentis Radix*, *Caulis Sinomenii*, *Cortex Periplocae*, *Radix Saposhnikoviae*, *Clematis chinensis*, *Atractylodes Lancea*, *Cinnamomum wilsonii Gamble*, *Cnidium monnieri*, *Alpinia officinarum*, *Foeniculum vulgare*, *Angelica sinensis*, *Radix Padoniae Rubra*, *Chaenomeles sinensis*, *Caesalpinia sappan* L., *Rheum officinale Baill.*, *Dipsacales Radix*, *Ligusticum chuanxiong hort*, *Angelica dahurica*, *Pini Lignum Nodi*, *Ephedra sinica Stapf*, *Boswellia carterii*, *Commiphora myrrh*, *Borneolum Syntheticum*, *Syzygium aromaticum*, *Cinnamomum cassia Presl*, and *Cinnamomum camphora*. All the above herbs were purchased from the Second Affiliated Hospital of Heilongjiang University of Chinese Medicine.

### 2.2 Sample preparation

#### 2.2.1 Preparation of self-made fried goupi plaster and blank matrix

According to 1/10 of the amount of goupi plaster in China Pharmacopoeia, 2020 [9]. Weighing 8g of *Radix Aconiti*, 4g of *Radix Aconiti Kusnezoffii*, 2g of *Notopterygium incisum*, 2g of *Angelicae Pubescentis Radix*, 3g of *Caulis Sinomenii*, 3g of *Cortex Periplocae*, 3g of *Radix Saposhnikoviae*, 3g of *Clematis chinensis*, 2g of *Atractylodes Lancea*, 2g of *Cnidium monnieri*, 3g of *Ephedra sinica Stapf*, 0.9g of *Alpinia officinarum*, 2g of *Foeniculum vulgare*, 1g of *Cinnamomum wilsonii Gamble*, 2g of *Angelica sinensis*, 3g of *Radix Padoniae Rubra*, 3g of *Chaenomeles sinensis*, 3g of *Caesalpinia sappan* L., 3g of *Rheum officinale Baill*, 3g of *Pini Lignum Nodi*, 4g of *Dipsacales Radix*, 3g of *Ligusticum chuanxiong hort* and 3g of *Angelica dahurica* respectively and break the medicinal materials into pieces. Fry the above medicines and 349.5g oil in the same pot, remove residues, filter and refine them into dripping medicinal oil. Add 114g of red lead (slightly fried without caking) into the oil, stir well (until it is removed in clear water and does not stick to hands), collect the paste, and soak it in water for seven days (change the water every day). Put 3.4g of *Boswellia carterii*, 3.4g of *Commiphora myrrh*, 1.7g of *Syzygium aromaticum*, 1.1g of *Cinnamomum cassia Presl*, 1.7g of *Borneolum Syntheticum*, 3.4g of *Cinnamomum camphora* are ground into powder. Melting the paste for removing fire poison with slow fire, adding the above powder, stirring, and spreading it on the backing, that is, the self-made fried Goupi plaster.

According to the dosage ratio of 3495:1140 in China Pharmacopoeia, 2020, soybean oil and red lead were weighed respectively [9]. Put the soybean oil into the pan, adjust the power of the induction cooker to 1200W and heat it for 10min. At this time, the oil temperature is about 220 °C, reduce the power of the induction cooker to 1000W, and continue heating for 30min until the oil temperature reaches about 320 °C. At this time, the smoke turns white and the dripping beads are observed. When the oil changes color and reaches the state of dripping beads, add the red lead into the oil, and keep stirring for 20∼25min. During this period, pay attention to observing the paste condition. When the paste does not stick to hands, it is cooled to about 200 °C from the fire, poured into water to cool and collect the paste, weighed and soaked in water to remove fire poison, and the water needs to be changed every day for 7 days to obtain the blank matrix of Goupi plaster.

#### 2.2.2 Preparation of goupi plaster using different extraction methods

Following the 2020 Chinese pharmacopoeia standards [9]. Chinese medicinal herbs that make up the fried part of Goupi plaster were crushed and placed into a 10 L extraction kettle, with double the amount required. For SFE-CO_2_ extraction, the kettle was filled with CO_2_ and set to 25 MPA and 45 °C. The first separation kettle was set to 8 MPA and 60 °C, and the second separation kettle was set to 4.5 MPA and 37 °C. The pump frequency was set at 18 Hz with a flow rate of 60 L/h and an extraction time of 3 h to obtain Goupi plaster through SFE-CO_2_ extraction [10]. For the ethanol extractions (95 %, 75 %, and 55 %) and water extraction, 1/10 quantity of Goupi plaster was soaked overnight with the corresponding solvent (12 times the quantity of Chinese medicinal herbs) and leached using a slow fire with four times of leaching for 2 h each time [11].

Five equal parts of the blank Goupi plaster base were gently heated and melted. The SFE-CO_2_ extract, 95 % ethanol extract, 75 % ethanol extract, 55 % ethanol extract, and water extract were then added. Subsequently, fine powders of *Boswellia carterii*, *Commiphora myrrh*, *Borneolum Syntheticum*, *Cinnamomum camphora*, *Syzygium aromaticum*, *Cinnamomum cassiaPresl* (ground through a 100-mesh sieve) were added and evenly mixed to making into corresponding Goupi plaster for later use.

#### 2.2.3 Preparation of control and test solutions

An appropriate amount of sinomenine, osthole, and cinnamaldehyde standard substances were accurately weighed and added to chromatographic methanol to prepare reference solutions with concentrations of 60.00 μg/mL, 100.00 μg/mL, and 100.00 μg/mL respectively [12].

To prepare the test solution, 10.00g of self-made Goupi plaster and Goupi plaster blank matrix were accurately weighed and evenly spread in separate beakers. Then, 30 mL of methanol solution was added to each beaker to ensure full contact with the plaster with the solvent. The mixture was ultrasonicated for 3 h and allowed to stand still. It was then placed in a refrigerator at −4 °C for 48h and filtered while cold. The solution was repeatedly filtered three times until it became oily, combine filtrates, recover under reduced pressure, add 10 mL chromatographic methanol to dissolve, and the filtrates were combined. Finally, the sample was filtered with a 0.45 μm filter membrane to obtain the test solution [12].

### 2.3 Chromatography and mass spectrometry conditions

For qualitative analysis, Thermo Vanquish UPLC and Q-Exactive HF high-resolution mass spectrometry were used [12]. Chromatographic separation was performed using a Zorbax Eclipse C18 column (100 mm × 2.1 mm, 1.8 μm). The column temperature was maintained at 30 °C. The mobile phase consisted of A (0.1 % formic acid water) and B (acetonitrile). The gradient profile was as follows: 0–2 min, 5 % B, 2–6 min, 30 % B, 6–7 min, 30 % B, 7–12 min, 78 % B, 12–14 min, 78 % B, 14–17 min, 95 % B, 17–20 min, 95 % B. The injection volume was 2 μL, the flow rate was 300 μL/min, and the autosampler temperature was set to 4 °C. For both positive and negative ion modes, the heater temperature was set to 325 °C, the sheath gas flow rate was 45 arbs, the auxiliary gas flow rate was 15 arbs, the purge gas flow rate was 1 arb, electrospray voltage was 3.5 KV, the capillary temperature was set to 330 °C, and the S-lensRF level was 55 %. The scan mode was *m*/*z* 100–1500, dd-MS2, TopN = 10; resolution: 120000 (MS1) & 60000 (MS2); and collision mode was High energy collision dissociation (HCD).

Quantitative analysis was conducted using a Waters UPLC I-Class/Xevo TQD system. Separation was performed on an ACQUITY UPLC C18 column at 30 °C. The mobile phase consisted of B (acetonitrile) and C (0.1 % formic acid water) [12]. The elution order for sinomenine and cinnamaldehyde mobile phase was as follows: 0–5 min, 10–95 % B; 5–10min, 95-10 % B; and 10–11min, 10-10 % B. Osthole mobile phase elution order was as follows: 0–2 min, 40-40 % B; 2–5 min, 40–90 % B; 5–6 min, 90-90 % B; and 6–8 min, 90-40 % B. The injection volume was 4 μL, and the flow rate was 0.20 mL/min.

### 2.4 Qualitative analysis in goupi plaster with different extraction methods

The test solutions mentioned above were analyzed using Compound Discoverer 3.2 software for retention time correction, peak identification, peak extraction, etc. Total ion current diagrams of Goupi plaster samples prepared by different extraction methods in positive and negative ion modes were obtained. Thermo mz Cloud online and Thermo mz Valut local databases were used to analyze secondary fragment ions, thus obtaining the retention time, molecular weight, accurate molecular weight deviation, and secondary fragment mass spectrum information of compounds, which helped in identifying the chemical substances.

Using the chromatographic peak retention time of the screened compounds as a variable, the principal component analysis was performed by importing SIMCA 14.1 software. This led to the creation of PCA score diagrams and Venn diagrams.

### 2.5 Method validation

The determination methodology was validated in accordance with the Chinese Pharmacopoeia 2020 [9].

#### 2.5.1 Linearity, limits of detection (LOD), and limits of quantiﬁcation (LOQ)

Reference solutions of sinomenine, osthole, and cinnamaldehyde were prepared and diluted proportionally with an appropriate amount of methanol. The concentration of sinomenine reference solution is 0.05 μg/mL, 1.00 μg/mL, 7.50 μg/mL, 15.00 μg/mL, 22.50 μg/mL, 60.00 μg/mL, the concentration of osthole reference solution is 0.10 μg/mL, 5.00 μg/mL, 10.00 μg/mL, 40.00 μg/mL, 70.00 μg/mL, 100.00 μg/mL, and cinnamaldehyde reference solution is 1.00 μg/mL、5.00 μg/mL、10.00 μg/mL、30.00 μg/mL、50.00 μg/mL、100.00 μg/mL. Shake well and pass through a 0.45 μm microporous filter membrane. Take the above-mentioned reference substances with different concentrations and inject them for determination. The standard curve of them was plotted with the reference concentration as the abscissa and the peak area as the ordinate, respectively.

The limit of detection (LOD) and the limit of quantification (LOQ) of each standard were determined when the signal-to-noise ratio (S/N) was 3 and 10, respectively.

#### 2.5.2 Precision, repeatability, stability, and sample recovery

Intra-day and inter-day precision changes of sinomenine, osthole, and cinnamaldehyde were evaluated to assess precision. Prepare sinomenine reference solution with three concentrations of 0.05 μg/mL, 7.50 μg/mL, 30.00 μg/mL, osthole reference solution with three concentrations of 0.10 μg/mL, 10.00 μg/mL, 100.00 μg/mL, cinnamaldehyde reference solution with three concentrations of 1.00 μg/mL, 10.00 μg/mL, 100.00 μg/mL respectively, and inject the standard solution with the above concentrations six times a day and continuously injecting them for three consecutive days to obtain the peak area, and calculate the concentration of the above solution according to the standard curve and calculate the RSD value.

To evaluate repeatability, 10.00g of Goupi plaster extracted with 95 % ethanol from the same batch was weighed, and the test solution was prepared. Samples were injected for determination, and the content and RSD value were calculated.

To determine the stability of the sample within 24 h, the sample solution of Goupi plaster extracted by 95 % ethanol was injected after 0, 4, 8, 16, and 24 h, and the peak area was obtained. The RSD value was calculated.

To calculate the recovery rate and RSD value, 1.00 mL of 95 % ethanol-extracted Goupi plaster sample solution was accurately sucked into a 5 mL volumetric flask. Sinomenine, osthole, and cinnamaldehyde reference solutions at low, middle, and high doses were added, respectively. Fill the volume with methanol, filtered, and sample injection. The recovery rate and RSD value were calculated according to the standard curve.

### 2.6 Quantitative analysis of sinomenine, osthole, and cinnamaldehyde

Three batches of Goupi plaster samples prepared by different extraction methods were used to measure the contents of index chemical components.

Chromatographic conditions from item 2.3. were used as the standard injection.

### 2.7 Data processing

Use Thermo mzCloud online and Thermo mzValut local databases to analyze secondary fragment ions. The retention time, molecular weight, deviation of the precise molecular weight and secondary fragment mass spectrometry information of the compound can be obtained. The analysis results are summarized and identified, and the Goupi plaster blank matrix group is removed. The chemical components in Goupi plaster with different extraction methods were obtained. Using the chromatographic peak retention time of the screened compounds as a variable, import it into SIMCA 14.1 software to perform principal component analysis and draw a PCA score plot. Origin 9.0 software was used for data processing and graphing (see Table 1).

**Table 1:** The mass spectrometry parameters for analytes.

Analytes	Parent ion(*m*/*z*)→product ion(*m*/*z*)	Lonization mode	Cone voltage(V)	Capillary voltage(KV)	Collision energy(V)
Sinomenine	330.30/151.10	ESI+	40.00	3.00	25.00
Osthole	244.86/189.00	ESI+	43.00	2.72	3.00
Cinnamaldehyde	133.20/115.10	ESI+	40.00	3.00	9.00

## 3 Results

### 3.1 Identification of chemical constituents

**Fig. 1:**
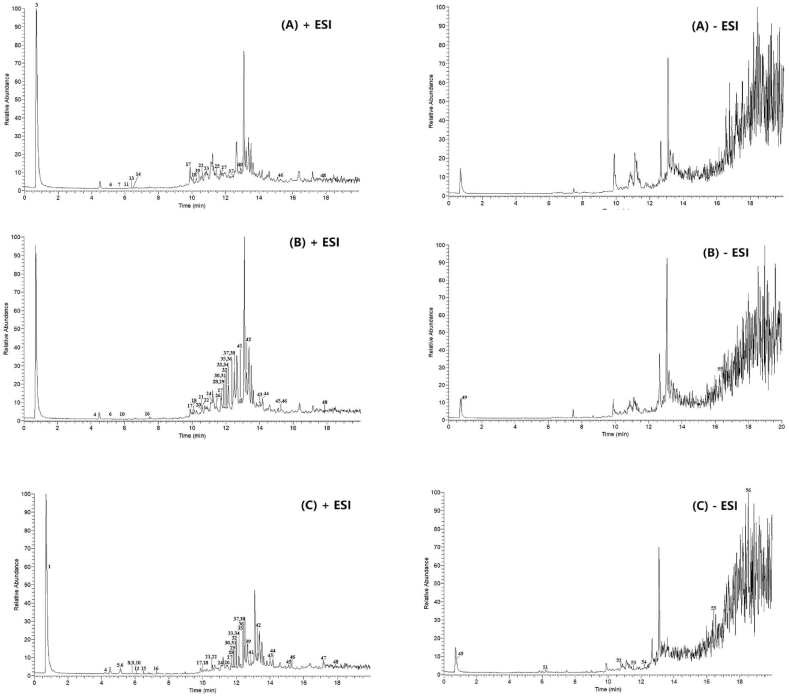

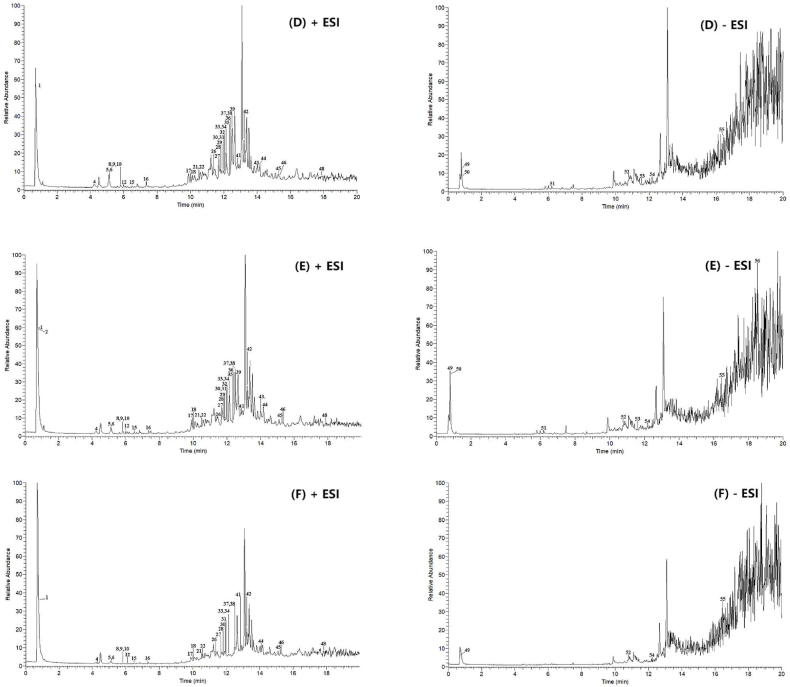
TIC diagram of Goupi plaster extracted using different extraction methods.(A: Fried extraction method B: SFE-CO_2_ extraction method C: 95 % ethanol extraction method D: 75 % ethanol extraction method E: 55 % ethanol extraction method F: Water extraction method).

The total ion current diagram (TIC) of Goupi plaster prepared by different extraction methods is shown in (Fig. 1). A total of 56 chemical components were screened from 6 Goupi plaster samples using different extraction methods, as shown in Table 2. Upon analysis, it was observed that the fried extraction group, SFE-CO_2_ extraction group, 95 % ethanol extraction group, 75 % ethanol extraction group, 55 % ethanol extraction group, and water extraction group contained 17, 33, 44, 42, 44, and 33 chemical components, respectively. Therefore, the order of quantity from most to least is 95 % ethanol extraction group = 55 % ethanol extraction group >75 % ethanol extraction group > water extraction group = SFE-CO_2_ extraction group > fried extraction group. Upon changing the extraction methods of Goupi plaster, the types of compounds in the samples changed significantly. The samples extracted by frying contained more compounds that were not found in other sample groups, which may be due to the low polarity of vegetable oil, incomplete extraction of the active ingredients in the drug, and the decomposition of compounds caused by high temperature. In the SFE-CO_2_ group, fewer chemical components were observed, possibly because SFE-CO_2_ is more suitable for extracting nonpolar or less polar components. In comparison, more chemical components were found in the ethanol extraction group than in the water extraction group. This may be because the effective components are more soluble in different concentrations of ethanol than in more polar water.

**Table 2:** Active components in Goupi plaster prepared by different extraction methods and their attribution.(Ⅰ).

NO.	ESI	Formula	Rt (min)	Theoretical Mass (*m*/*z*)	Experimental Mass (*m*/*z*)	Compound [Ref.]	Main fragment ions (*m*/*z*)	Error (ppm)	Adduct	Attribution
1	+	C5H11NO2	0.783	117.0793	117.0789	Betaine [13]	118.086, 59.073, 58.065	3.45	[M+H]+	C,D,E,F
2	+	C12H22O11	0.786	342.1168	342.1162	Maltose	381.08, 203.053	1.61	[M+H]+	E
3	+	C6H7NO	0.822	109.0532	109.0527	3-Hydroxy-2-methylpyridine	110.060, 82.065, 53.997	4.29	[M+H]+	A
4	+	C10H15NO	4.228	165.1157	165.1154	Pseudoephedrine [14]	166.123, 148.112, 91.054	2.21	[M+H]+	B,C,D,E,F
5	+	C22H31NO3	5.113	357.2307	357.2304	Oxybutynin [15]	358.238, 72.081, 58.065	0.91	[M+H]+	C,D,E,F
6	+	C19H23NO4	5.124	329.163	329.1627	Sinomenine [16]	330.170, 270.149, 159.696	1.01	[M+H]+	A,B,C,D,E,F
7	+	C25H41NO7	5.758	467.2892	467.2883	Lycoctonine	468.231, 436.212	2.07	[M+H]+	A
8	+	C10H12O4	5.805	196.074	196.0736	Cantharidin	197.085, 127.042, 91.053	2.37	[M+H]+	C,D,E,F
9	+	C16H22O9	5.806	358.127	358.1264	Sweroside	197.080, 127.049	1.66	[M+H]+	C,D,E,F
10	+	C20H23NO4	5.839	341.1634	341.1627	6- acetylcodeine [17]	342.170, 297.113, 265.097	2.05	[M+H]+	B,C,D,E,F
11	+	C22H33NO3	6.068	359.2463	359.246	Cyclomethycaine	360.215, 328.242	0.82	[M+H]+	A
12	+	C22H28O11	6.095	468.1642	468.1631	Prim-O-glucosylcimifugin	469.170, 307.128	2.31	[M+H]+	C,D,E,F
13	+	C26H37N5O2	6.352	451.2947	451.2938	6-Allyl-N-[3-(dimethylamino)propyl]-N-(ethylcarbamoyl)ergoline-8-carboxamide	452.331, 420.215	−1.85	[M+H]+	A
14	+	C24H35NO5	6.487	417.2523	417.2515	Decoquinate	418.259, 385.20, 222.040	2.09	[M+H]+	A
15	+	C21H25NO4	6.497	355.1789	355.1783	Glaucine	365.19, 279.10, 152.062	1.78	[M+H]+	C,D,E,F
16	+	C31H43NO10	7.356	589.2899	589.2887	Benzoylmesaconine [18]	590.297, 240.260	2.07	[M+H]+	B,C,D,E,F
17	+	C9H8O	9.997	132.0578	132.0575	Cinnamaldehyde [19]	133.065, 115.055, 95.050	2.79	[M+H]+	A,B,C,D,E,F
18	+	C12H8O4	10.096	216.0426	216.0423	8-methoxypsoralen [20]	217.050, 202.026, 89.039	1.67	[M+H]+	A,B,C,D,E,F
19	+	C15H20O2	10.443	232.1465	232.1463	Atractylenolide II [21]	232.146, 215.161	0.89	[M+H]+	A
20	+	C15H16O4	10.543	260.1052	260.1049	Isomeranzin [22]	261.131, 189.055	1.31	[M+H]+	B

21	+	C10H10O2	10.545	162.0683	162.068	4-Methoxycinnamaldehyde	163.075, 135.082, 50.015	1.79	[M+H]+	B,C,D,E,F
22	+	C13H10O5	10.591	246.0531	246.0528	Isopimpinellin [20]	247.060, 232.037	1.27	[M+H]+	A,B,C,D,E,F
23	+	C13H10O5	10.897	246.0530	246.0528	Pimpinellin	247.061, 232.049	0.78	[M+H]+	A
24	+	C17H16O6	11.167	316.0952	316.0947	Byakangelicol [23]	317.325, 233.045, 231.029	1.48	[M+H]+	B,C
25	+	C10H16O	11.406	152.1203	152.1201	(−)-Camphor [24]	153.127, 109.065, 65.039	1.51	[M+H]+	A
26	+	C8H8O	11.411	120.0579	120.0575	Acetophenone	121.065, 103.055, 93.071	3.4	[M+H]+	B,C,D,E,F
27	+	C12H16O2	11.722	192.1154	192.115	Senkyunolide A [25]	193.123, 175.112	2.11	[M+H]+	A,B,C,D,E,F
28	+	C20H32O2	11.822	304.2405	304.2402	Mesterolone [26]	305.248, 287.237,135.117	0.72	[M+H]+	B,C,D,E,F
29	+	C12H14O2	11.856	190.0997	190.0994	3-*n*-Butylphathlide	191.107, 173.096, 145.101	1.69	[M+H]+	B,C,D,E
30	+	C11H14O4	11.973	210.0896	210.0892	Methylxanthoxylin	211.097, 193.086	1.64	[M+H]+	B,C,D,E,F
31	+	C15H24O	11.992	220.1831	220.1827	(−)-Caryophyllene oxide [24]	221.190, 203.180, 135.117	1.63	[M+H]+	B,C,D,E,F
32	+	C12H12O2	12.030	188.0841	188.0837	n-Butylidenephthalide [27]	189.091, 133.029	1.95	[M+H]+	B,C,D,E
33	+	C16H14O4	12.150	270.0895	270.0892	Imperatorin [9]	271.242, 203.034, 147.044	1.00	[M+H]+	B,C,D,E,F
34	+	C11H6O4	12.151	202.0269	202.0266	Bergaptol [28]	203.034, 89.043	1.46	[M+H]+	B,C,D,E,F
35	+	C12H18O2	12.393	194.131	194.1307	Neocnidilide	195.138, 149.133	1.71	[M+H]+	B,C,D,E
36	+	C17H16O5	12.423	300.1001	300.0998	Phellopterin [29]	301.107, 233.044, 62.015	1.07	[M+H]+	B,C,D,E
37	+	C15H16O3	12.496	244.1102	244.1099	Osthole [30]	245.117, 189.055, 131.049	1.36	[M+H]+	A,B,C,D,E,F
38	+	C11H8O3	12.503	188.0477	188.0473	Plumbagin	189.055, 131.049	1.75	[M+H]+	B,C,D,E,F
39	+	C16H14O4	12.685	270.0895	270.0892	Isoimperatorin [9]	271.096, 203.034, 65.039	1.00	[M+H]+	B,C,D,E
40	+	C20H26O3	12.853	314.1881	314.1881	Kahweol	315.196, 165.070, 91.054	−0.21	[M+H]+	A
41	+	C14H12O3	12.855	228.079	228.0786	Psoralen [20]	229.086, 187.039, 141.701	1.49	[M+H]+	B,C,D,E,F
42	+	C18H30O2	13.229	278.2249	278.2246	α-keto acid	279.232, 132.081	0.86	[M+H]+	B,C,D,E,F
43	+	C24H28O4	14.074	380.1992	380.1988	Levistilide A	381.280, 191.107	1.20	[M+H]+	B,C,D,E
44	+	C21H34O3	14.179	334.251	334.2508	Siegesmethyetheric acid	335.258, 317.248	0.74	[M+H]+	B,C,D,E,F

45	+	C30H46O3	15.232	454.3553	454.3447	Oleanonic acid [31]	455.352, 109.102	1.45	[M+H]+	B,C,D,E,F
46	+	C30H46O4	15.277	470.3403	470.3396	Glycyrrhetic acid	471.347, 271.206, 91.054	1.75	[M+H]+	A,B,C,D,E,F
47	+	C6H12N4	17.191	140.1064	140.1062	Methenamine	141.113, 112.087, 85.076	1.79	[M+H]+	C
48	+	C32H48O5	17.873	512.3511	512.3501	3-Acetyl-11-keto-β-boswellic acid [9]	513.358, 287.213	1.82	[M+H]+	A,B,C,D,E,F

NO.	ESI	Formula	Rt (min)	Theoretical Mass (*m*/*z*)	Experimental Mass (*m*/*z*)	Compound [Ref.]	Main fragment ions (*m*/*z*)	Error (ppm)	Adduct	Attribution
1	–	C12H22O11	0.788	342.1168	342.1162	Trehalose [13]	341.109, 179.056, 89.024	−0.04	[M − H]-	B,C,D,E,F
2	–	C18H32O16	0.791	504.1691	504.169	d-Raffinose	527.158, 163.03	0.13	[M − H]-	D,E
3	–	C23H28O11	6.193	480.1635	480.1631	Paeoniflorin [9]	479.121, 121.029	0.73	[M − H]-	C,D,E
4	–	C14H14O3	10.788	230.0942	230.0939	7-Demethylsuberosin [32]	229.087, 174.015	−1.46	[M − H]-	C,D,E,F
5	–	C16H12O5	11.575	284.0685	284.0684	Glycitein [33]	283.161, 268.038	0.09	[M − H]-	C,D,E
6	–	C15H10O5	12.204	270.0529	270.0528	Genistein	269.046, 217.055, 133.029	0.38	[M − H]-	C,D,E,F
7	–	C30H48O3	16.455	456.3607	456.3603	Oleanolic acid [9]	455.353, 216.311	0.85	[M − H]-	B,C,D,E,F
8	–	C4H6O4	18.522	118.0269	118.0266	Succinic acid [34]	116.928, 90.009, 73.030	2.92	[M − H]-	C,E

#### 3.1.1 PCA analysis

**Fig. 2:**
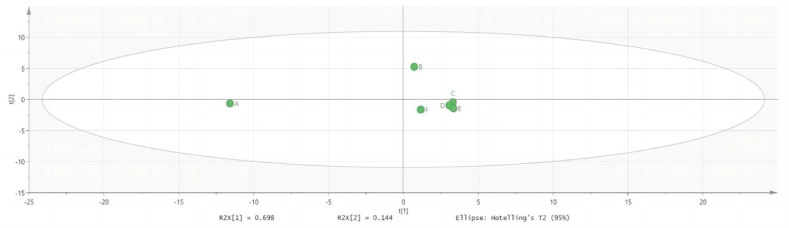
PCA score plot of Goupi plaster prepared using different extraction methods.

**Fig. 3:**
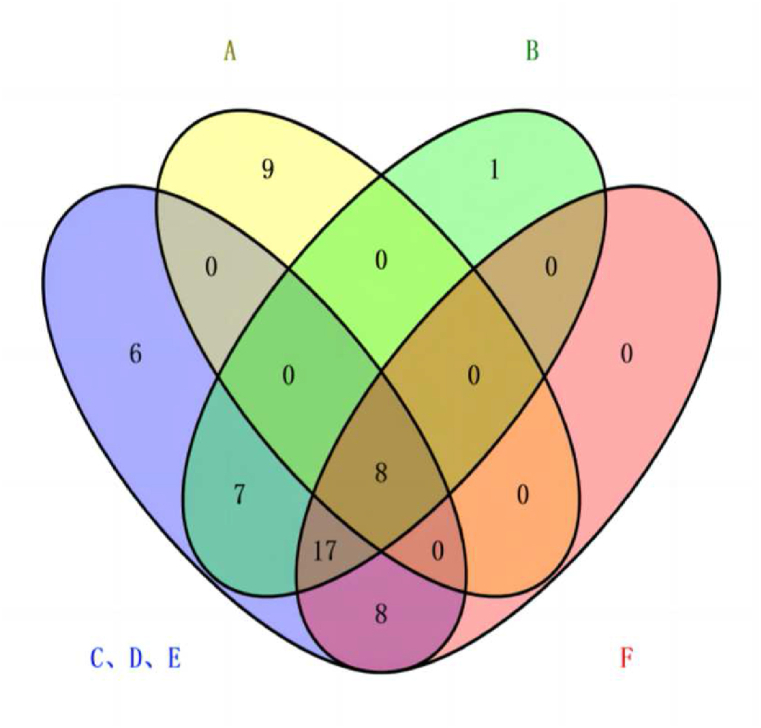
Venn diagram showing the chemical composition of Goupi plaster prepared using different extraction methods.

As shown in (Fig. 2), the SFE-CO_2_ extraction group was distributed in the first quadrant, indicating that there were differences in chemical composition compared to the other groups. The 95 % ethanol extraction group, 75 % ethanol extraction group, and 55 % ethanol extraction group, which had similar components, were put together and compared with the fried extraction group, SFE-CO_2_ extraction group, and water extraction group. Among them, there were 8 completely overlapping parts and 48 non-overlapping differences, as shown in the Venn diagram in (Fig. 3). In the overlapping part, effective components such as sinomenine, cinnamaldehyde, and osthole which have been shown to treat KOA, were found in all groups. Among the non-overlapping differences, components with higher LogP values were found to be more lipophilic, such as isohesperelactone (LogP: 1.88), Angelica sinensis brain (LogP: 2.05), and neosnake-bed lactone (LogP: 2.83) in the SFE-CO_2_ group. These components have pharmacological effects, such as anti-inflammatory, antibacterial, anti-tumor, and analgesic properties. Conversely, components with lower LogP values were found to be more hydrophilic, such as betaine (LogP: 3.25), maltose (LogP: 3.41), and cantharidin (LogP: 0.1), which all appeared in the ethanol or water extraction groups. These components have pharmacological effects, including liver protection, lipid metabolism regulation, and anti-inflammation. In terms of solubility, swertiamarin, cimicifuginoside, new snake bed lactone, coralline, and isoimperatorin are all soluble in ethanol. Therefore, these components appear in different ethanol extraction groups, and they have pharmacological effects such as analgesia, anti-inflammatory, antipyretic, antithrombotic, liver protection, antibacterial and osteoporosis prevention. In summary, the chemical compositions of samples from the ethanol extraction groups (C, D, E) were similar, indicating that the concentration of ethanol had little effect on the chemical compositions. However, these samples differed from those obtained from the fried extraction group (A), SFE-CO_2_ extraction group (B), and water extraction group (F). This suggested that the polarity of the extraction methods may be one of the reasons for these differences.

#### 3.1.2 Sinomenine fragmentation pathway

**Fig. 4:**
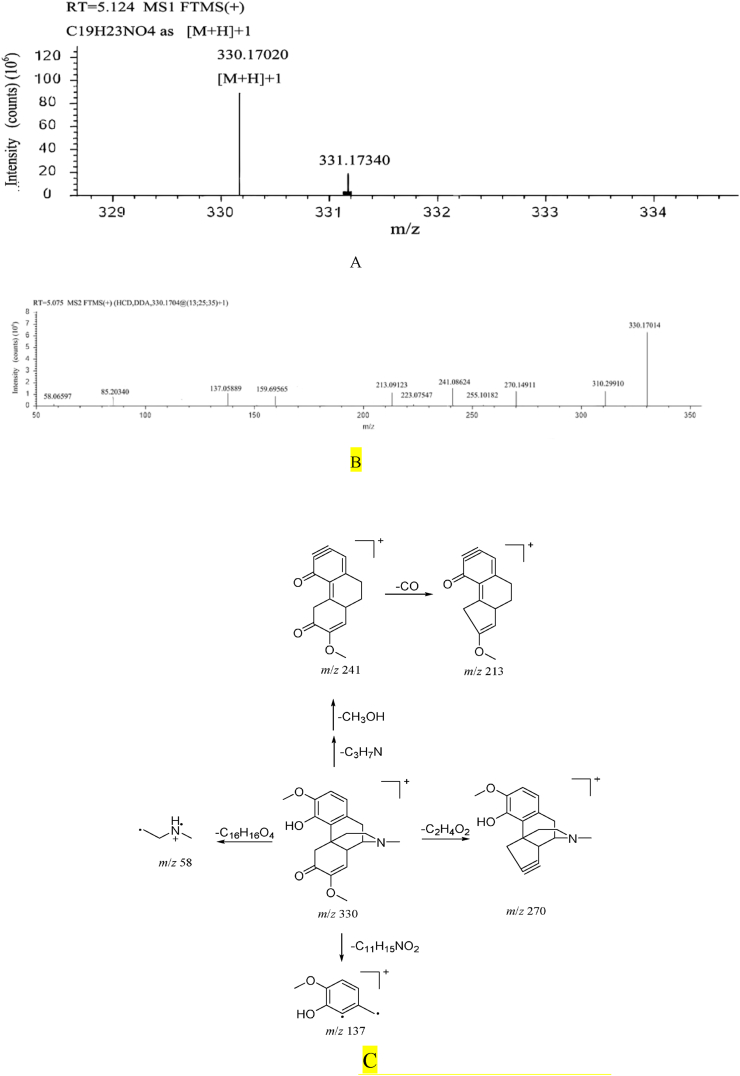
A. Primary mass spectrum of sinomenine B. Secondary mass spectrum of sinomenine C. The fragmentation pathway of sinomenine.

Sinomenine, retention time t_R_ = 5.12 min. Quasi-molecular ion *m/z* 330.17020 [M+H]+, molecular formula of which is preliminarily estimated to be C_19_H_23_NO_4._ It is easy to lose the tertiary amine (CH_2_CH_2_NCH_3_), and at the same time, the removed tertiary amine will combine with protons to generate secondary fragment ion *m/z* 58 (CH_2_CH_2_NH ^+^ CH_3_). The secondary fragmentation ions are *m/z* 270 [M-H-C_2_H_4_O_2_]^+^, *m/z* 241 [M-H–C_3_H_7_N–CH_3_OH]^+^, *m/z* 213 [M-H–C_3_H_7_N–CH_3_OH–CO]^+^, *m/z* 137 [M-H-C_11_H_15_NO_2_]^+^, *m/z* 58 [M-H-C_16_H_16_O_4_]^+^. Combined with the fragmentation law of mass spectrometry and literature reference [35,36]. It is inferred that the compound is sinomenine. (Fig. 4).

#### 3.1.3 Osthole fragmentation pathway

**Fig. 5:**
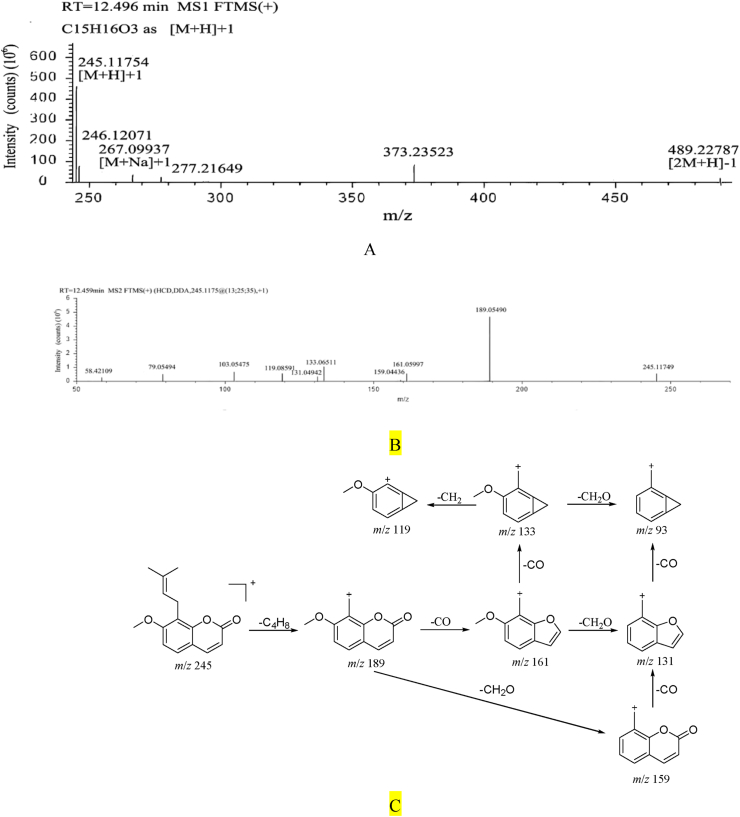
A. Primary mass spectrum of osthole B. Secondary mass spectrum of osthole C. The fragmentation pathway of osthole.

Osthole, retention time t_R_ = 12.50 min. Quasi-molecular ion *m*/*z* 245.11754 [M+H]+, the molecular formula is preliminarily estimated to be C_15_H_16_O_3_. The cracking law is that isobutenyl (C_4_H_8_) is firstly cracked and then neutral micromolecules such as CO and CH_2_O are removed. The secondary cracking fragment ions include *m/z* 189 [M-H-C_4_H_8_]^+^, *m/z* 161 [M-H–C_4_H_8_–CO]^+^, *m/z* 159 [M-H–C_4_H_8_–CH_2_O]^+^, *m/z* 133 [M-H–C_4_H_8_–2CO]^+^, *m/z* 131 [M-H–C_4_H_8_–CO–CH_2_O]^+^, *m/z* 119 [M-H–C_4_H_8_–2CO–CH_2_]^+^, *m/z* 93 [M-H–C_4_H_8_–2CO–CH_2_O]^+^. Combined with the fragmentation law of mass spectrometry and literature reference [37]. It is inferred that the compound is osthole. (Fig. 5).

#### 3.1.4 Cinnamaldehyde fragmentation pathway

**Fig. 6:**
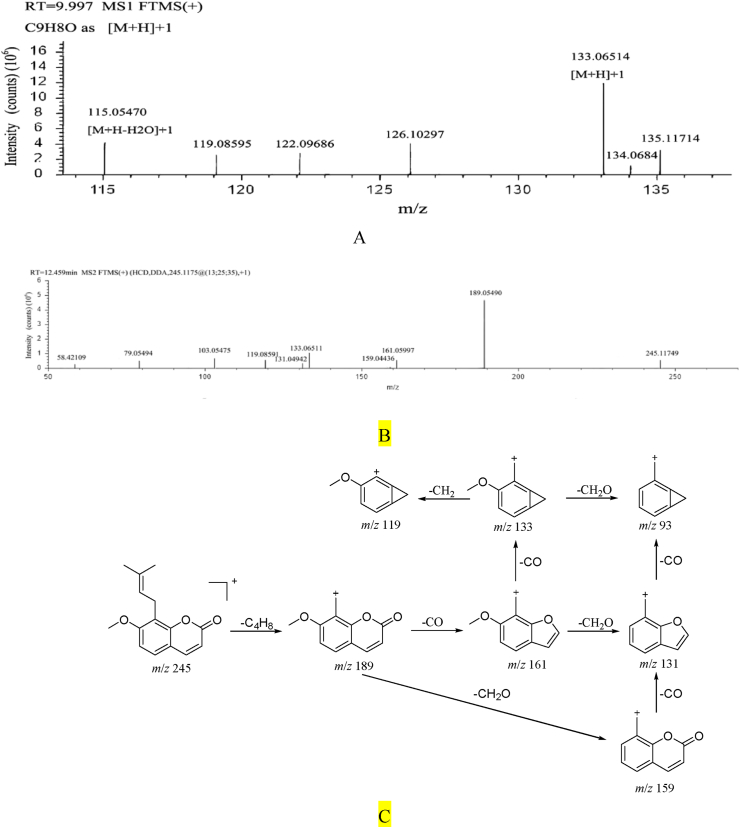
A. Primary mass spectrum of cinnamaldehyde B. Secondary mass spectrum of cinnamaldehyde C. The fragmentation pathway of cinnamaldehyde.

Cinnamaldehyde, retention time t_R_ = 9.99 min. Quasi-molecular ion *m*/*z* 133.06514 [M+H]+, molecular formula C_9_H_8_O. The secondary cracking fragment ions include *m/z* 115 [M-H-H_2_O]^+^, *m/z* 105 [M-H-CO]^+^, *m/z* 103 [M-H–H_2_O–C]^+^, *m/z* 91 [M-H–H_2_O–2C]^+^. Combined with the fragmentation law of mass spectrometry and literature reference [38]. It is inferred that the compound is cinnamaldehyde. (Fig. 6).

### 3.2 Method validation for quantitative assay

#### 3.2.1 Specificity

**Fig. 7:**
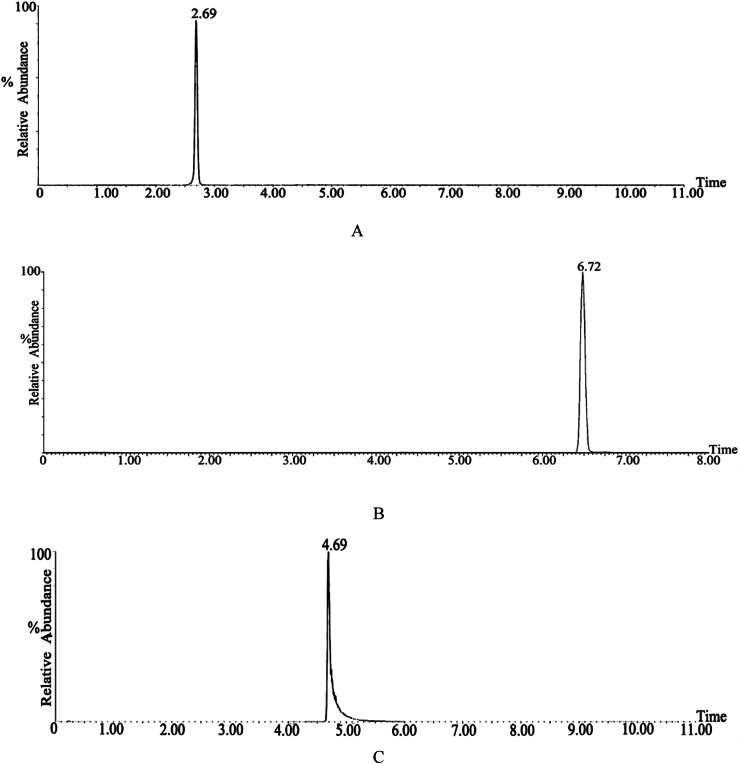
The total ion flow diagram for specificity investigation.(A. Total ion flow diagram of sinomenine standard B. Total ion flow diagram of osthole standard E. Total ion flow diagram of blank matrix in Goupi plaster C. Total ion flow diagram of cinnamaldehyde standard).

Fig. 7 chromatogram shows that no interference from other impurity peaks at the peak position, with good peak shapes and resolution. The retention times for sinomenine, osthole, and cinnamaldehyde were 2.69 min, 6.72 min, and 4.69 min, respectively, indicating that the method had excellent specificity.

#### 3.2.2 Linearity, limits of detection (LOD), and limits of quantiﬁcation (LOQ)

Table 3 shows that the following chemical components exhibit a good linear relationship within the corresponding concentration range. The regression equation, correlation coefficient, linear range, LOD, and LOQ values are listed (see Table 4).

**Table 3:** The regression equation, linear range, limits of detection, limits of quantification of three index components in Goupi plaster.

Analytes	Calibration curves	R^2^	Linear range (μg/mL)	LOD (ng/mL)	LOQ (ng/mL)
Sinomenine	y = 8401.5x + 319.59	0.9993	0.05–60.00	0.20	0.65
Osthole	y = 2122.7x + 3416.8	0.9991	0.10–100.00	0.03	0.13
Cinnamaldehyde	y = 789.56x-379.08	0.9993	1.00–100.00	0.05	0.17

**Table 4:** Contents of three index components in Goupi plaster.

Peer group	Sinomenine μg/g	Osthole μg/g	Cinnamaldehyde μg/g
Fried extraction	0.29 ± 0.01	0.13 ± 0.01	10.52 ± 0.01
SFE-CO_2_ extraction	0.27 ± 0.02	82.33 ± 0.09	50.10 ± 0.53
95 % ethanol extraction	25.97 ± 0.02	26.83 ± 0.01	27.10 ± 0.19
75 % ethanol extraction	21.07 ± 0.01	23.78 ± 0.01	26.75 ± 0.22
55 % ethanol extraction	7.02 ± 0.01	22.18 ± 0.02	19.16 ± 0.32
Water extraction	6.04 ± 0.01	2.62 ± 0.01	34.18 ± 0.21

#### 3.2.3 Precision, stability, repeatability, and sample recovery rate

The intra-day and inter-day precision RSD of sinomenine ranged from 0.10 % to 1.85 %, while that of osthole was between 0.19 % and 0.56 %, and that of cinnamaldehyde ranged from 0.92 % to 1.90 %. These results suggest that the system is stable and has good precision. The RSD values of sinomenine, osthole, and cinnamaldehyde in the stability test were less than 0.94 %, 1.66 %, and 0.31 % respectively, while in the repeatability test, the RSD values of sinomenine, osthole, and cinnamaldehyde were less than 0.88 %, 0.30 %, and 0.18 % respectively. These results indicate that goupi plaster was stable within 24 h, and the system had good repeatability. The recoveries of sinomenine, osthole, and cinnamaldehyde were between 96.89 %–101.35 %, 97.32 %–101.68 %, and 96.43 %–100.89 %, respectively, The relative standard deviations were 1.35 %, 1.51 %, and 1.32 %, respectively, all of which were less than 2.00 %. These results demonstrate that the recovery rate of this method was good.

### 3.3 Quantitative analysis

Significant variations were observed in the levels of sinomenine, cinnamaldehyde, and osthole in Goupi plaster extracted using six different solvents. For the content of sinomenine was in the following order: 95 % ethanol extraction >75 % ethanol extraction >55 % ethanol extraction > water extraction > fried extraction > SFE-CO_2_ extraction. Regarding the content of cinnamaldehyde was in the following order: SFE-CO_2_ extraction > water extraction >95 % ethanol extraction >75 % ethanol extraction >55 % ethanol extraction > fried extraction.Concerning the content of osthole was in the following order: SFE-CO_2_ extraction >95 % ethanol extraction >75 % ethanol extraction >55 % ethanol extraction > water extraction > fried extraction. Analysis of the cumulative content of these three constituents revealed that the Goupi plaster extracted with SFE-CO_2_ has the highest content. Although the cinnamaldehyde and osthole levels were elevated in this sample, the sinomenine content was relatively lower than in other group. The Goupi plaster extracted with 95 % ethanol ranked second in the total content of the three compounds, with balanced contents of sinomenine, cinnamaldehyde and osthole. The other Goupi plaster sample groups had higher levels of these three components compared to the fried extraction samples, further indicating that a loss of active ingredients occurs during the traditional Goupi plaster preparation through frying.

## 4 Discussion

### 4.1 The effect of extraction methods on the types of compounds

Up to 29 kinds of Chinese herbal medicines exist in Goupi plaster. Several chemical components of Goupi plaster with different extraction methods have been identified in databases and related literature. It was found that the change of extraction methods had different extraction effects on the effective components of Goupi plaster, and the extracted chemical components of other groups exist certain differences compared with fried extraction group, which may be the result of the combined action of extraction methods and the polarity of extraction solvents. Notably, due to its special preparation method, and the high temperature in the process of frying extraction may lead to the oxidation polymerization of the original compounds and generate some new chemical component, such as 6-Allyl-N-[3-(dimethylamino)propyl]-N-(ethylcarbamoyl)ergoline-8-carboxamide, Cyclomethycaine and other compounds and secondary products. Alternatively, this phenomenon could stem from the low polarity of vegetable oil, which might fail to fully extract the active constituents in crude drugs and may lead to compound decomposition due to high temperature. SFE-CO_2_ extraction targets nonpolar or slightly polar components, typically utilized for extracting volatile oil components, thereby enabling it to extract more coumarins compared to water reflux extraction [39]. Moreover, samples extracted with 55 % ethanol contain a similar variety of compounds to those extracted with 95 % ethanol. It is also likely that 55 % ethanol can extract both ethanol-soluble and water-soluble substances. Due to the presence of polysaccharides and tannins in the medicinal herbs, these substances were found in large quantities in water extraction and 55 % ethanol extraction. By excluding substances with unknown pharmacological effects, samples extracted using 95 % ethanol contained the most types of active components. Therefore, we conclude that the ethanol extraction method has certain advantages.

### 4.2 Selection of index components

The composition of Goupi plaster is complex, and its active components include alkaloids, coumarins, phenolic acids and volatile oils. However, the effective components in Goupi plaster prepared by the traditional method may be fewer. This calls for the search for representative active substances in the traditional preparation. There are a lot of active alkaloids in *Radix Aconiti*, *Radix Aconiti Kusnezoffii* and *Caulis Sinomenii.* However, through preliminary experimental exploration,it is found that aconitine is not detected in the fried Goupi plaster samples, therefore, sinomenine in *Caulis Sinomenii* can be used as an index component for alkaloid extraction and evaluation in Goupi plaster. Sinomenine possesses anti-inflammatory and immunomodulatory properties. Research indicates that sinomenine can strongly alleviate joint swelling symptoms in rats and ameliorate inflammatory cell infiltration, synovial cell congestion, fibrous tissue proliferation, bone destruction, and other associated changes [40]. *Cinnamomum cassia Presl* in Goupi plaster has the effect of warming meridians, dredging collaterals and relieving pain, and their effective components are mainly cinnamaldehydes. Studies have found that medium and high concentrations of cinnamaldehyde can significantly inhibit the proliferation activity of KOA inflammatory synovial cells [41].Therefore, cinnamaldehyde can be used as an index component for the extraction and evaluation of volatile oil in Goupi plaster. Various types of coumarins are also found in Goupi plaster, such as *Radix Angelicae Pubescentis*, *Fructus Cnidii*, *Radix Angelicae Sinensis* and *Radix Saposhnikoviae*. Its representative component is osthole. Osthole possess anti-inflammatory and analgesic pharmacological effects. Related studies have found that Osthole can significantly inhibit the translocation of p65 and the activity of nuclear factor κB in rat aortic endothelial cells induced by angiotensin II, thereby decrease the expression of inflammatory cytokines such as TNF-α, IL-6 and IL-1β [42]. Therefore, building on prior investigations into the shared chemical constituents of Goupi plaster across various manufacturers [12]. Selecting sinomenine, cinnamaldehyde and osthole as extraction evaluation indexes of Goupi plaster with different extraction solvents can allow comprehensive assessment of the extraction efficacy of alkaloids, coumarins and volatile oils present in Goupi plaster with different extraction solvents.

## 5 Conclusion

In this study, UPLC-Q-Exactive-MS technology was used to rapidly and accurately identify the chemical components in Goupi plaster extracted by different solvents. Results indicated that the chemical components in the samples significantly change after changing the extraction method of Goupi plaster. The fried extraction group, SFE-CO_2_ extraction group, 95 % ethanol extraction group, 75 % ethanol extraction group, 55 % ethanol extraction group and water extraction group contained 17,33,44,42,44 and 33 chemical components respectively. The content determination methods of sinomenine, osthole and cinnamaldehyde in Goupi plaster were established by UPLC-MS/MS technology, and the results of method validation all met the requirements. The linear relationships of sinomenine, osthole and cinnamaldehyde were good in the concentration ranges of 0.05–60.00 μg/mL, 0.10–100.00 μg/mL and 1.00–100.00 μg/mL, respectively. Significant differences were recorded in the contents of sinomenine, cinnamaldehyde and osthole in the Goupi plaster prepared by six different solvents. The content of sinomenine was in the range of 0.2713–25.9665 μg/g, the content of sinomenine in 95 % ethanol extraction group was the highest, and lowest in the SFE-CO_2_ extraction group. The cinnamaldehyde content ranged from 10.5202 to 50.0969 μg/g, with the SFE-CO_2_ extraction group exhibiting the highest levels and the fried extraction group displaying the lowest. Meanwhile, osthole content varied between 0.1329 and 82.3345 μg/g, with the SFE-CO_2_ extraction group recording the highest content and the fried extraction group yielding the lowest. The contents of sinomenine, cinnamaldehyde and osthole in 95 % ethanol extraction group were balanced, and the contents of these three components in Goupi plaster extracted by other different solvents were higher than those in Goupi plaster extracted by frying. The above results demonstrate that changing the extraction method of Goupi plaster may induce significant variations in chemical components. Considering the diverse Chinese herbal medicines in Goupi plaster and their complex mechanism of drug action, further studies should aim to solve the retention problem of fried extraction of Goupi plaster. This study serves a valuable reference for the scientific exploration of the fried extraction method of Goupi plaster, and is also of great significance for the improvement of dosage forms and the research and development of new dosage forms of Goupi plaster in the future.

## Funding statement

This study was supported by the 10.13039/501100001809National Natural Science Foundation of China (Grant approval No. 82074025) and Heilongjiang Touyan Innovation Team Program.

## Data availability statement

Data will be made available on request.

## Ethics declarations

Review and/or approval by an ethics committee was not needed for this study because this work was not involve ethical considerations in animal, cell and human experiments.

## CRediT authorship contribution statement

**Tong Guan:** Writing – review & editing, Writing – original draft, Visualization, Methodology, Investigation, Conceptualization. **Rong Wang:** Methodology, Investigation, Formal analysis, Data curation. **Jiajing Wang:** Investigation, Data curation. **Qingqing Zhang:** Investigation, Conceptualization. **Ziheng Liu:** Validation, Investigation. **Zhixin Yang:** Resources, Conceptualization. **Feng Guan:** Supervision, Methodology. **Weinan Li:** Resources, Data curation. **Yanhong Wang:** Supervision, Project administration, Methodology, Funding acquisition.

## Declaration of competing interest

The authors declare that they have no known competing financial interests or personal relationships that could have appeared to influence the work reported in this paper.
